# Reduction of dielectric artifacts within an InSightec ExAblate 4000 head transducer

**DOI:** 10.1186/2050-5736-3-S1-P27

**Published:** 2015-06-30

**Authors:** Steve Leung, Pejman Ghanouni, Kim Butts Pauly

**Affiliations:** 1 Stanford University, Stanford, California, United States

## Background/introduction

High intensity focused ultrasound (HIFU) ablation of the brain has been increasingly used as a non-invasive treatment for essential tremor and neuropathic pain. However, artifacts arise in magnetic resonance (MR) images as a result of dielectric properties of the water bath coupler and of the tissues being investigated. As radio frequency (RF) waves enter a dielectric medium, their wavelengths decrease by a factor equal to the square root of the medium’s relative permittivity (Webb 2011). The shortened wavelength can cause a standing wave pattern within the sample, with regions of constructive and destructive interference. This RF inhomogeneity results in dielectric artifacts that appear as shaded regions in the intensity image. Modifying the RF coil can be used to bypass this issue, but is non-trivial. We present a simpler solution by decreasing the permittivity of the water bath, thereby reducing the standing wave effect that creates dielectric artifacts.

## Methods

To decrease the permittivity of the water bath, sodium chloride (NaCl) was dissolved at concentrations of 12.5 to 62.5 mM at 12.5 mM increments. Higher concentrations correlated with lower permittivity. A fast gradient echo (FGRE) T1W sequence (TR 250, TE 13.2, FOV 40 cm x 40 cm, slice thickness 5 mm, 256 x 128 matrix) was performed on a 3T GE scanner. A region of interest (ROI) was drawn at the center of the head transducer along the superior/inferior direction. Signal to noise ratio (SNR) was calculated, in which signal was the mean intensity across the ROI and noise was the standard deviation of background.

## Results and conclusions

**Figure 1:**
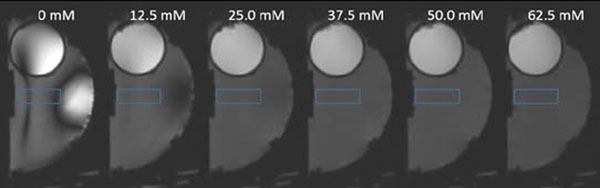
Side-by-side comparison of intensity images at different concentrations. Images show a sagittal view of an InSightec ExAblate 4000 head transducer with a water bath and head phantom.

**Figure 2:**
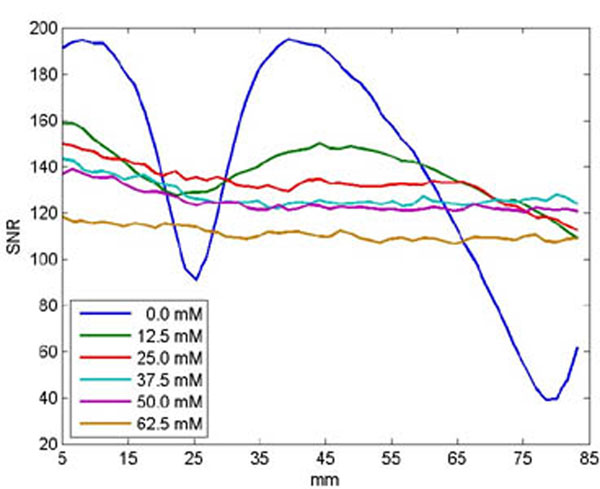
SNR profile along the length of the ROI. The distance on the x-axis is the distance from the geometric focus. No signal was measured from 0 to 5 mm due to the Plexiglas protrusion into the water bath.

Varying the concentration of NaCl had a noticeable effect on the dielectric artifact (Figure 1). Qualitatively, the artifact was significantly reduced at a concentration of 37.5 mM. At higher concentrations, the images look relatively homogenous. The SNR profiles along the rectangular ROI are given in Figure 2. Due to dielectric artifacts, images at concentrations of 0, 12.5, and 25.0 mM displayed SNR inhomogeneity. In comparison, images at concentrations of 37.5 mM and above were much more homogenous. Higher concentrations showed a gradual loss of signal due to reduction of the dielectric effect. Salts such as NaCl act as corrosive agents, therefore it is best to limit its use with electrical equipment. The optimal concentration appears to be 37.5 mM, due to the fact Figure 2. SNR profile along the length of the ROI. The distance on the x-axis is the distance from the geometric focus. No signal was measured from 0 to 5 mm due to the Plexiglas protrusion into the water bath. Salts such as NaCl act as corrosive agents, therefore it is best to limit its use with electrical equipment. The optimal concentration appears to be 37.5 mM, due to the fact that it best balances the tradeoffs between signal homogeneity, SNR, and salt concentration. This study presents a simple and inexpensive solution to reduce dielectric artifacts seen in MR images when using an InSightec ExAblate 4000 head transducer for non-invasive treatment. The reduction of dielectric artifacts will allow for ease of image diagnosis and analysis.

